# Supplementary material for: Empirical models for anatomical and physiological changes in a human mother and fetus during pregnancy and gestation
Source: PLoS One. 2019 May 2;14(5):e0215906. doi: 10.1371/journal.pone.0215906 (PMC6497258; doi:10.1371/journal.pone.0215906)
Supplement: S1 File — (PDF) [file pone.0215906.s001.pdf]

Model Calibration Details

Supplement to "Empirical models for anatomical and physiological changes in a human mother and fetus during pregnancy and gestation" by Dustin F. Kapraun1, John F. Wambaugh, R. Woodrow Setzer, Richard S. Judson

In considering any particular model and any given quantity of interest y (e.g., a maternal body mass), we used data to identify the model parameters $\{\theta_0, \theta_1, \dots\}$ such that y can be estimated as a function of an independent variable t (e.g., gestational age or body mass) and said parameters. The parameters were identified using maximum likelihood estimation as described below.

For each quantity of interest, we obtained a data set of the form $\mathcal{D} = \{(t_i, y_i, \sigma_i, n_i)\}_{i=1}^{m}$, where y_i, σ_i, and n_i represent the mean, standard deviation, and number (or sample size) of the values observed for the quantity of interest at time t_i. The symbol m represents the total number of time points. For some data sets, each observed mean value y_i for the quantity of interest was paired with a corresponding mass x_i (e.g., body mass) instead of a time t_i, but the model calibration proceeded in the same way in either scenario. Hereafter in this section, we let t_i denote a time or a mass as appropriate for the data set in question. Also, for some data sets, only ordered pairs $\{(t_i, y_i)\}_{i=1}^{m}$ were available (i.e., standard deviations and samples sizes $\{\sigma_i, n_i\}_{i=1}^{m}$ were not available). In these cases, we assumed $\sigma_i = 0.2 \cdot (\sum_{i=1}^{m} y_i)/m$ (i.e., 20% of the mean data value) and $n_i = 1$ for all i so that all ordered pairs in such data sets were weighted equally when calibrating. While the default value of 20% coefficient of variation was chosen somewhat arbitrarily, it should be noted that this choice does not affect the optimal parameters identified through maximum likelihood estimation; it does, however, effect the ultimate likelihood function values, and therefore impacts the selection of an optimal model.

Model calibration involves finding a set of k parameters given by $\theta = (\theta_0, \theta_1, \dots, \theta_{k-1})$ such that an algebraic model given by $y(t; \theta)$ (cf. Table 2 of main manuscript) achieves the "best" possible fit to the

data $\mathcal{D}$; that is, the goal is to find θ such that $y(t_i;\theta)$ is "close" to y_i for each data point i. We chose the best set of parameters for a given model and data set using maximum likelihood estimation (see, e.g., [1]); i.e., for each model we found the set of parameters that maximizes the value of a likelihood function.

To construct a likelihood function, we assumed that the differences between model predictions and observed data are normally distributed. Thus, we make use of the probability density function for a normally distributed random variable $X \sim N(\mu, \sigma)$ in the discussion below. This density function is given by

$$f_X(x;\mu,\sigma) = (2\pi\sigma^2)^{-1/2} \cdot \exp\left[\frac{-(x-\mu)^2}{2\sigma^2}\right],$$

where x denotes the observed value of the random variable X, and μ and σ denote the mean and standard deviation, respectively, of the normal distribution describing X. For our purposes, we assumed each y_i to be a realization of a random variable $Y_i \sim N(y(t_i;\theta), \sigma_i)$. Maximum likelihood thus requires that $y(t_i;\theta)$ be closer to y_i when σ_i is smaller (i.e., for those data points for which we have greater confidence or lower variability in the observed value of the quantity of interest). Based on the aforementioned assumption, our likelihood function is given by

$$\mathcal{L}(\theta;\mathcal{D}) = \prod_{i=1}^{m}\prod_{j=1}^{n_i} f_Y(y_i; y(t_i;\theta), \sigma_i)$$

$$= \prod_{i=1}^{m}\prod_{j=1}^{n_i} (2\pi\sigma_i^2)^{-1/2} \cdot \exp\left[\frac{-(y_i - y(t_i;\theta))^2}{2\sigma_i^2}\right].$$

In practice we work with the logarithm of the likelihood function, or log-likelihood function, which is given by

$$\ell(\theta; \mathcal{D}) = \sum_{i=1}^{m} n_i \left[-\frac{1}{2}\log(2\pi) - \log(\sigma_i) - \frac{1}{2}\frac{\big(y_i - y(t_i;\theta)\big)^2}{\sigma_i^2} \right].$$

Because the logarithm function is strictly increasing, the value of θ that maximizes the likelihood function also maximizes the log-likelihood function. This optimal value of θ, which we denote $\hat{\theta}$, is called the maximum likelihood estimate (MLE). It is worth noting that the MLE is equivalent to the weighted least squares estimate of θ (see, e.g., [2]) in which each data point (t_i, y_i) is assigned a weight equal to n_i/σ_i^2.

References

1. Casella G, Berger RL (2002) Statistical Inference. Pacific Grove, CA: Duxbury.
2. Bradley EL (1973) The Equivalence of Maximum Likelihood and Weighted Least Squares Estimates in the Exponential Family. Journal of the American Statistical Association 68: 199-200.
